# C Proteins: Controllers of Orderly Paramyxovirus Replication and of the Innate Immune Response

**DOI:** 10.3390/v14010137

**Published:** 2022-01-12

**Authors:** Oliver Siering, Roberto Cattaneo, Christian K. Pfaller

**Affiliations:** 1 Division of Veterinary Medicine, Paul-Ehrlich-Institute, 63225 Langen, Germany; Oliver.Siering@pei.de; 2 Department of Molecular Medicine, Mayo Clinic, Rochester, MN 55906, USA

**Keywords:** *Paramyxoviridae*, *Orthoparamyxovirinae*, replication, nucleocapsid, processivity, defective-interfering RNA, immune evasion, inflammasome, budding, ESCRT

## Abstract

Particles of many paramyxoviruses include small amounts of proteins with a molecular weight of about 20 kDa. These proteins, termed “C”, are basic, have low amino acid homology and some secondary structure conservation. C proteins are encoded in alternative reading frames of the phosphoprotein gene. Some viruses express nested sets of C proteins that exert their functions in different locations: In the nucleus, they interfere with cellular transcription factors that elicit innate immune responses; in the cytoplasm, they associate with viral ribonucleocapsids and control polymerase processivity and orderly replication, thereby minimizing the activation of innate immunity. In addition, certain C proteins can directly bind to, and interfere with the function of, several cytoplasmic proteins required for interferon induction, interferon signaling and inflammation. Some C proteins are also required for efficient virus particle assembly and budding. C-deficient viruses can be grown in certain transformed cell lines but are not pathogenic in natural hosts. C proteins affect the same host functions as other phosphoprotein gene-encoded proteins named V but use different strategies for this purpose. Multiple independent systems to counteract host defenses may ensure efficient immune evasion and facilitate virus adaptation to new hosts and tissue environments.

## 1. Introduction

About 50 years ago, when the proteins from purified particles of different paramyxoviruses were first analyzed, several polypeptides were identified and their biological roles tentatively attributed [1,2,3,4,5]. Three proteins (nucleocapsid, polymerase and phosphoprotein) are now known to constitute the replicative complex, and three others (attachment, fusion and matrix proteins) constitute the membrane fusion apparatus [6]. Another particle-associated protein with a molecular weight (MW) of about 42.000, labeled A, turned out to be cellular actin [7].

When the synthesis of virus-specific proteins was analyzed in infected cells, two proteins not found in particles, and thus deemed nonstructural, were identified [7]. These proteins had MWs of about 36,000 and 20,000, respectively, and were labeled with the letters B and C. Alternatively, the MW 36,000 protein was labeled with the Roman numeral V (five) that was sometimes pronounced as the letter V.

When these two proteins, currently named V and C, were shown to be minor virus particle components, the nonstructural nomenclature was dropped. When reverse genetics showed that V and C are not essential for virus replication, they were reclassified as “accessory” [8,9,10,11]. However, even this classification is misleading, because both V and C are required for the productive infection of natural hosts [12,13,14,15]. We review here the multiple functions of the paramyxovirus C proteins in the control of virus replication and of the host innate immune response and propose that C should stand for “control”.

## 2. C Proteins Are Expressed from Alternative Open Reading Frames in the P/V/C Genes

**Figure 1:**
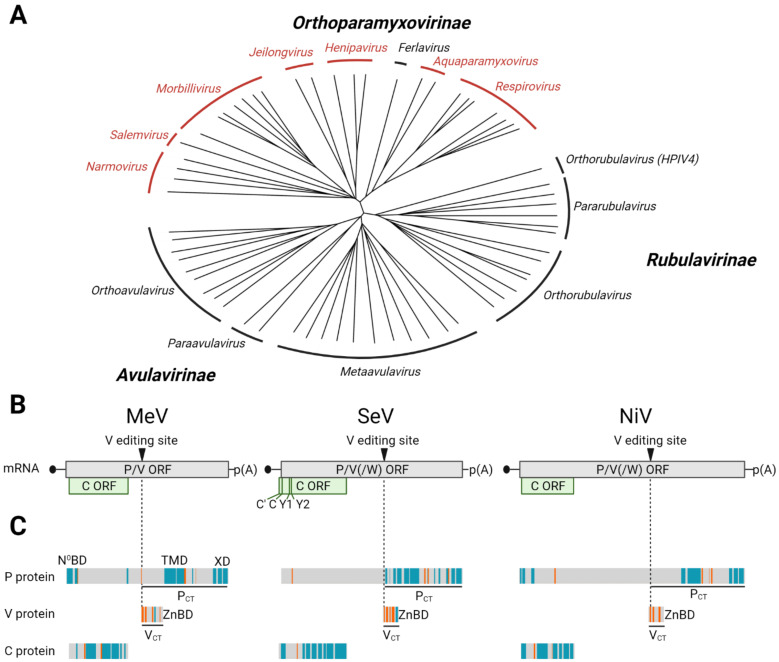
C protein expression among viruses of the *Paramyxoviridae* family. (**A**) Phylogenetic tree indicating subfamilies and genera. The tree is based on full genome alignment of available reference genomes in GenBank using CLUSTALW [18] and was generated with iTOL [19]. Viruses of genera in red express C proteins. (**B**) Organization of the overlapping open reading frames in the mRNAs derived from the P/V/C genes of MeV (**left**), SeV (**center**) and NiV (**right**). SeV expresses four C proteins from alternative start codons named C’, C, Y1 and Y2. (**C**) Secondary structure predictions for P and C proteins, as well as the V protein-specific carboxy-terminus (V_CT_). Alpha-helices are depicted in blue, beta strands in orange and unstructured (coiled) regions are shown in grey. Underlined regions in P and V proteins correspond to their unique carboxy-termini (P_CT_ and V_CT_). N^0^BD: binding domain interacting with free nucleoprotein; TMD: tetramerization domain of P; XD: domain of P interacting with N_tail_ of the nucleocapsid; ZnBD: Zn-binding domain of V protein that interacts with several host innate immunity factors. Figure generated with BioRender.com.

Paramyxoviruses (Figure 1A) have non-segmented RNA genomes of negative polarity that are 15–20 kilobases in length. Most mRNAs transcribed from these templates code for a single protein product, but early analyses of Sendai virus (SeV) phosphoprotein (P) mRNA suggested it might encode the C protein in addition to the P protein [16]. Indeed, the SeV P mRNA nucleotide sequence revealed two overlapping reading frames. The first encodes the 588-amino acid P protein; the second starts at an AUG located 10 nucleotides downstream of the P AUG and codes for the 204-amino acid C protein [17] (Figure 1B, top center).

Successive analyses of the measles virus (MeV) P gene revealed a similar gene organization: the MeV C AUG is located 22 nucleotides downstream of the P AUG [20] (Figure 1B, top left). Initially, no sequence homology was detected between the SeV and MeV C proteins. A third type of C proteins was discovered when the P gene of the newly emerged Nipah virus (NiV) was sequenced [21] (Figure 1B, top right). The NiV C AUG is located 23 nucleotides downstream of the P AUG, putting it in the “+2” reading frame, while both the SeV and MeV C reading frames are “+1”, as compared to the respective P reading frames.

C proteins, or candidate C protein reading frames, are common but not ubiquitous in paramyxoviruses; they have been characterized in 26 of the 63 genomic reference sequences currently available in GenBank (Figure 1A; paramyxovirus genera expressing C proteins are shown in red and Table 1). On the other hand, almost every paramyxovirus P gene encodes a third product, the V protein. V proteins consist of the amino-terminal half of P fused to a carboxyl-terminal domain highly conserved in the *Paramyxoviridae* that includes seven invariant cysteines [22,23] (Figure 1C, center). In different paramyxoviruses, V proteins are either produced by a frame shift introduced by RNA editing or from unedited RNA, while the P expression depends on editing [6]. Even if the V and C proteins have completely different sequences and structures, they can accomplish similar tasks by operating through different mechanisms [24]. V proteins are particularly important for virus genera that lack C proteins, while genera that express both proteins have the option to counteract host defenses through alternative strategies in different cells and tissues. Our review will focus on the functions of C proteins. The different functions of V proteins have been comprehensively reviewed elsewhere [24,25,26,27,28].

## 3. All Three Types of C Proteins Include an Intrinsically Disordered Part

**Figure 2:**
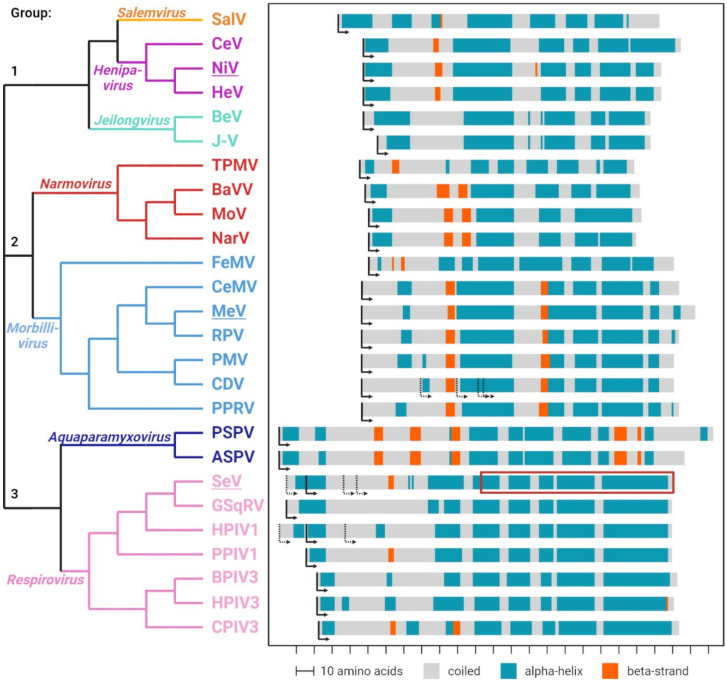
Sequence and structure comparison of paramyxoviral C proteins. Phylogenetic analysis of the C protein sequences (**left**). Different genera are highlighted in individual colors. Prototype species underlined. Sequences were aligned using the PROMALS3D tool [56], and a phylogenetic analysis was done via the neighbor-joining clustering method using the EMBL-EBI Simple Phylogeny tool [57]. Predicted secondary structures of the different C proteins (**right**). Alpha-helices are depicted in blue, beta strands in orange and unstructured (coiled) regions are shown in grey. Translation initiation codons are indicated by arrows. Predictions were performed with the JPred tool [58]. The red box indicates the region in the SeV C protein for which a tertiary structure is available (PDB 3WWT) [59]. Figure generated with BioRender.com.

While the primary amino acid conservation of C proteins is very low, it is sufficient to categorize them into three distinct groups [55] (Figure 2, left). Proteins from the *Henipavirus*, *Salemvirus* and *Jeilongvirus* genera form group 1; proteins from the *Morbillivirus* and *Narmovirus* genera form group 2 and proteins from the *Respirovirus* and *Aquaparamyxovirus* genera form group 3 (Figure 2, top to bottom).

Secondary structure predictions indicate that the carboxy-terminal part of all C proteins is rich in alpha-helixes, whereas little secondary structures are predicted for the amino-terminal part, which appears to be intrinsically disordered (Figure 2, right). The C proteins of groups 1 and 2 show high similarities in their predicted secondary structures, whereas group 3 C proteins are more distant from the other two groups.

Notably, when the P and the C reading frames overlap, only one of them is predicted to fold into alpha-helixes or beta-strands, whereas the other appears to be intrinsically disordered (Figure 1C), a characteristic of dynamic protein structures [60]. Intrinsically disordered segments may be required for the P protein to walk the polymerase down the ribonucleocapsids (RNPs) during replication and transcription and may allow C to interact with several different cellular partners.

## 4. Functional Insights from the SeV C-STAT1 Complex Structure

The structures of intrinsically disordered proteins are difficult to determine, but complexes of parts of the SeV C protein (amino acids 98–204) with two different cellular partners have been crystallized. In one complex, the C protein associates with the signal transducer and activator of transcription 1 (STAT1), a transcription factor involved in the interferon (IFN) response (PDB 3WWT) [59]. The second partner is the ALG2-interacting protein X (ALIX), a component of the endosomal sorting complex required for transport (ESCRT) (PDB 6KP3) [61].

**Figure 3:**
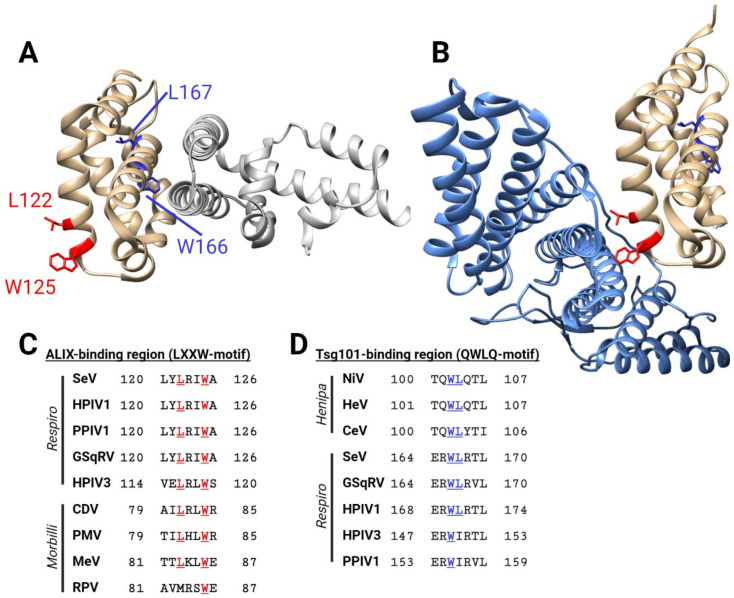
Structural features of the SeV C protein. (**A**) SeV C protein (amino acid residues 99–204, shown in gold) in a complex with STAT1 (silver) (PDB 3WWT) [59]. Residues important for binding to ALIX (LXXW motif) are shown in red; potential Tsg101-binding residues (QWLQ-motif) are shown in blue. (**B**) SeV C protein (amino acid residues 99–204, shown in gold) in a complex with the BRO1 domain of ALIX (light blue) (PDB 6KP3) [61]. (**C**) Conserved ALIX-binding motifs in respiroviruses (top five sequences), and homologous residues in morbilliviruses (bottom four sequences). (**D**) Conserved Tsg101-binding motifs in henipaviruses (top three sequences), and homologous residues in respiroviruses (bottom 5 sequences). Figure generated with BioRender.com.

Figure 3A provides a lateral view of the SeV C-STAT1 complex, illustrating the compact globular folding of the C alpha-helical domain (Figure 3A, gold), which may be similar in other C proteins. Notably, STAT1 and ALIX bind to opposite surfaces of the SeV C protein (Figure 3A,B). Several functions of the C proteins have been mapped to the alpha-helical carboxy-terminal half, which is consistent with the suggestion that this intrinsically disordered part either binds different proteins or controls the access of different partners to the alpha-helical part.

Several lines of evidence support this assumption. First, SeV and HPIV1 use several alternative start codons within the intrinsically disordered segment for the translation initiation of elongated (C’) or truncated (Y1, Y2) C proteins [62,63]; even the smallest so far detected C protein isoform Y2 retains the majority of its biological functions [64]. More-over, the morbillivirus CDV also generates C proteins with multiple alternative start codons, but only a recombinant CDV unable to express all C proteins is strongly attenuated in vitro and in vivo [65]. Finally, biochemical studies on artificially truncated C proteins of SeV, HPIV3 and MeV suggest that their amino terminus is not essential for C protein functions on regulation of viral replication or innate immunity control [66,67,68].

## 5. C Proteins Are Basic and Shuttle between Cytoplasm and Nucleus

Small proteins (<25 kDa molecular weight) can traverse the nuclear pore complex by diffusion [69]. Since the molecular weights of C proteins range from 17 to 27 kDa, it is not surprising that initial studies reported both the cytoplasmic and nuclear localization of MeV and SeV C proteins [20,70]. In contrast, a report suggested that the NiV C protein is exclusively localized in the cytoplasm [71]. More recent mechanistic analyses revealed that MeV, SeV and NiV C proteins all shuttle between the cytoplasm and nucleus.

In particular, the MeV C protein sequence ^75^DLEKAMTTLKLWE^87^ includes a nuclear export signal (NES) [D/E/Q]X_0–1_[L/I/M]X_2–3_[L/I/V/M/F]X_2–3_[L/M/V/F]X[L/M/I/V]X_0–3_[D/E] [72], and MeV C may interact with the chromosomal region maintenance 1 nuclear export protein (CRM1; also known as exportin 1, XPO1). Moreover, a basic amino acid stretch in this protein amino terminal region (^41^PPARKRRQ^48^) serves as the monopartite nuclear localization signal (NLS) for nuclear import by importin-α/β [72,73]. Thus, NES and NLS sequences allow nucleocytoplasmic shuttling of the MeV C protein [72,73].

For SeV and NiV C proteins, nonclassical NES and NLS that may act independently of CRM1 or importin-α/β have been identified [74,75]. Notably, the location of these sequences within the C proteins of different genera is not conserved, suggesting independent evolution. The independent evolution of nucleocytoplasmic shuttling implies relevance for the viral replication cycle, even if these viruses replicate only in the cytoplasm. By interfering with the nuclear import/export machineries, C proteins may regulate the cellular localization of host factors or the export of host mRNAs from the nucleus [76].

## 6. C Proteins Enhance Virulence through Multiple Mechanisms

**Figure 4:**
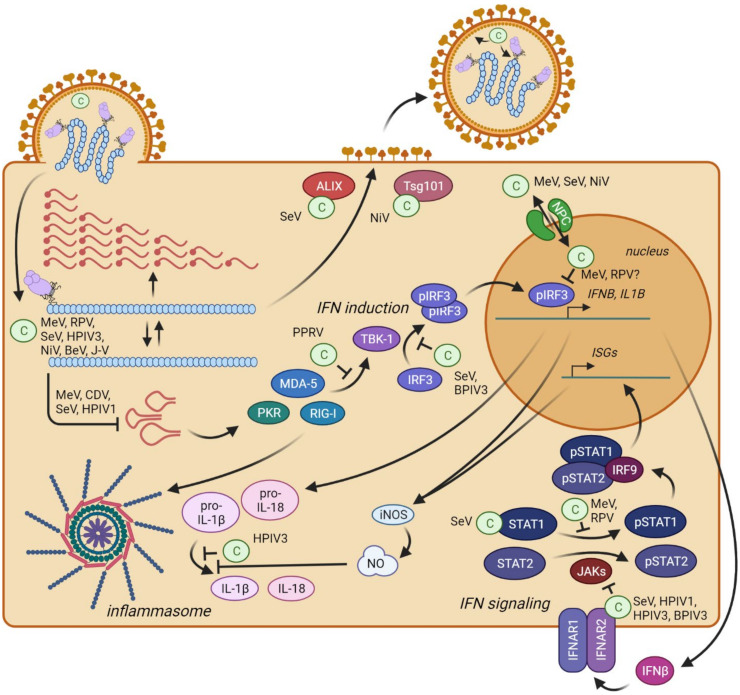
Overview of the localization and functions of the C protein in the paramyxovirus life cycle, and antagonism of the innate immunity pathways involved in paramyxovirus sensing. (**Top left**) After cell entry, the viral polymerase transcribes mRNAs and replicates the viral genome. C proteins regulate these processes and prevent the formation of immunostimulatory DI genomes (panhandle structures). (**Center**) Type-I IFN (IFNβ) induction after the sensing of DI genomes by cellular receptors PKR, RIG-I and MDA-5. C proteins can interfere with these processes by blocking the signal transduction cascade at multiple steps in the cytoplasm and nucleus. (**Bottom right**) Type-I IFN signaling activates the JAK/STAT pathways involving STAT1 and STAT2. The SeV C protein binds and sequesters STAT1; the other C proteins inhibit STAT1 phosphorylation. (**Bottom left**) The HPIV3 C protein also blocks the inflammasome-mediated activation of IL1β. (**Top center**) The C proteins interact with ESCRT components to enhance virus particle assembly and budding. SeV C interacts with ALIX, and NiV C interacts with Tsg101. (**Top right**) Several C proteins shuttle between the cytoplasm and nucleus utilizing the nuclear pore complex (NPC). Figure generated with BioRender.com.

In the nucleus or cytoplasm, C proteins have evolved several different functions. These include the regulation of viral transcription and replication, enhancement of viral polymerase processivity, control of the innate immune response and support of particle assembly and budding (Figure 4). Some of these functions are conserved across the paramyxovirus genera, and others are characteristic of one genus.

The development of reverse genetics technology for mononegaviruses [77,78] was instrumental for the identification and characterization of the different C protein functions. Genetic deletion of the C ORF from the MeV strain Edmonston B revealed that this protein is not required for MeV replication in IFN-defective Vero cells [9,79,80]. Similarly, C-deficient respiroviruses, henipaviruses and other morbilliviruses are replication-competent in certain transformed cell lines [81,82,83,84] but replicate less efficiently than wild-type viruses in other cell lines or in primary cells [13,65,85,86,87].

Hence, C proteins are essential antagonists of innate immune responses, a conclusion consistent with the finding that C-deficient viruses generally show a high degree of attenuation in vivo [13,15,50,65,82,84,88,89,90]. For respiroviruses and morbilliviruses, C-deficient mutant viruses exhibit strongly reduced pathogenesis in their natural hosts or animal models [15,65,82]. A C protein-deficient NiV was attenuated in vitro and showed reduced pathogenesis in two different animal models: hamsters and ferrets [91,92]. However, in both cases, the virus could still cause a fatal disease, and in the ferrets, the disease progression was only marginally altered compared to wild-type NiV infection [92]. Taken together, these findings indicate that paramyxoviral C proteins are virulence factors. However, how do they work?

## 7. C Proteins Regulate Viral Transcription and Replication

Analyses of SeV transcription suggested that its C proteins regulate viral RNA synthesis. The addition of C proteins to a SeV in vitro transcription system reduces the amount of transcribed viral mRNA [93], but C-deficient or partially C-defective SeV produce more mRNA than wild-type SeV [94]. A C-deficient SeV exhibits an altered genome-to–antigenome ratio [95], possibly due to promotor selectivity [96]. In particular, in wild-type SeV particles and infected cells, there are about five times more genomes than antigenomes [95], but this ratio changes with SeV deficient in C’ and C proteins [94,95,97]. Thus, the C protein may control genome or antigenome synthesis, dictating negative genome polarity by promoting the initiation of RNA synthesis from the antigenomic trailer sequence [97].

Another effect of the C protein is to alter the ratios of different viral RNA species in infected cells [98,99,100]. In particular, a C-deficient MeV exhibits a steeper transcription gradient than its parental virus [101]. C-deficient Nipah, HPIV3, J and Beilong viruses also have different transcription alterations [102,103,104,105,106]. Altered ratios of the viral RNA species can affect the production of progeny viruses either directly by delaying specific replication processes or indirectly due to the enhanced induction of innate immunity. Notably, C-deficient SeV, HPIV3, MeV and CDV caused enhanced innate immunity activation [65,101,107,108,109,110].

## 8. C Proteins Minimize Production of Immunostimulatory DI RNA

Innate immunity can be activated by viral double-stranded RNA (dsRNA) accidentally generated during viral replication. DsRNA can activate the cytoplasmic pattern recognition receptors that include the RNA sensors retinoic acid inducible gene-I (RIG-I), melanoma differentiation associated gene 5 (MDA-5) and the double-stranded (ds) RNA-dependent protein kinase (PKR). These sensors induce the transcriptional upregulation of type-I IFN (Figure 4, IFN induction).

In infections with C-deficient SeV, HPIV1, MeV and CDV, large amounts of copy-back defective interfering RNAs (DI RNAs) have been detected [101,107,110], and their structures have been characterized [65,101,111,112]. DI RNAs arise when the viral polymerase randomly detaches from the template RNA strand during replication. The polymerase, still bound to the newly synthetized RNA fragment, reinitiates synthesis using the nascent RNA strand as the new template [101,113,114]. Consequently, the newly generated RNA molecule is a hybrid of (−)- and (+)-strand RNA with complementary 5′ and 3′ ends. These ends can form double-stranded panhandles that efficiently activate dsRNA sensors RIG-I, MDA-5 and PKR (Figure 4), contributing to virus attenuation [65,101,110,111,112].

## 9. C proteins Interact with the Polymerase Complex

**Figure 5:**
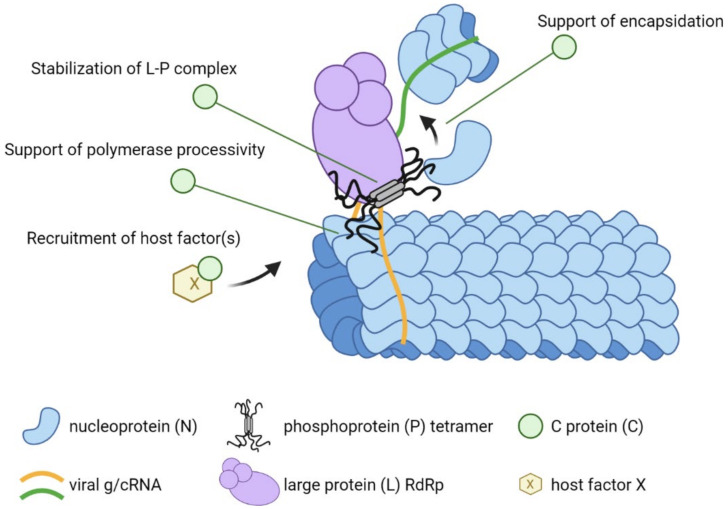
Schematic representation of the replicating paramyxovirus RNP and potential roles of C proteins in enhancing polymerase processivity. The viral nucleocapsid consists of RNA (orange curved line) encapsidated with N (blue shapes). The viral polymerase consisting of L (purple) and tetrameric P (grey) extracts viral RNA from the nucleocapsid and synthesizes a complementary sequence (green curved line). P moves the polymerase along the genomic RNA by sequentially interacting with helically arranged N subunits. P also interacts with free N to encapsidate the nascent RNA strand. C proteins may enhance polymerase processivity through one or several of the four indicated mechanisms. Figure generated with BioRender.com.

Thus, C proteins minimize the production of DI RNAs, but how? The mechanisms under investigation include different interaction partners or interaction sequences for different viruses (Figure 5). For SeV C, a direct interaction with L was initially proposed. It was observed that the SeV C protein colocalizes with the RNP and that this association is resistant to treatment with detergents [115]. The direct association of SeV C and L is consistent with the results of pull-down assays [116]. Other studies conducted with the closely related HPIV3 and the morbillivirus RPV are consistent with direct or indirect C–L interactions [117,118]. The interaction site of SeV C with L was mapped to several charged amino acid carboxy-terminal domains [119], and it was found that the C–L and P–L interaction sites do not overlap [120,121]. The precise understanding of how SeV C interacts with the polymerase complex awaits high-resolution structural analyses that are not yet available, but some considerations can be done based on the available high-resolution structure of the polymerase of another negative-strand RNA virus [122]. By binding to L, SeV C may simply stabilize the L–P complex or rearrange the individual L domains in specific configurations, thereby affecting mRNA synthesis and genome/antigenome replication.

In contrast to SeV C, the MeV C protein interacts initially with the N and P proteins [68,123]. This suggests other potential action mechanisms (Figure 5). By interacting with P and N, the MeV C protein may influence the encapsidation process of the nascent RNA strand. Improper encapsidation may lead to stalling of the viral polymerase and, eventually, the premature termination of RNA synthesis. Alternatively, MeV C may directly stabilize the interaction of the polymerase complex with the template RNP and secure the proper movement of the polymerase along the RNP. As shown for SeV C, absence of the MeV C protein may result in the premature termination of RNA synthesis. Notably, the alpha-helical carboxy-terminus of the MeV C protein is essential and sufficient for its interaction with P [68].

Interestingly, recent studies have identified several host factors that are recruited to paramyxoviral replication centers to support viral RNA synthesis [124,125,126,127]. The MeV C protein specifically recruits host factor SHCBP1 to the viral polymerase complex, which had a positive effect on viral replication [127]. It is likely that C proteins of other paramyxoviruses may also recruit host factors supporting viral replication (Figure 5).

In summary, paramyxoviral C proteins are integral parts of the viral replication complex, controlling polymerase activity and thereby ensuring proper RNA synthesis. Although the C proteins from individual genera may act through different molecular mechanisms, the control of replication is likely a major function that affects the virulence and pathogenesis of all C protein-expressing paramyxoviruses.

## 10. C Proteins Directly Interfere with Innate Immunity Activation

C proteins can directly block the innate immune system at multiple levels (Figure 4). Together with the V proteins [24,25,26,27,28,128], C proteins interfere with IFN induction and signaling and also prevent inflammatory responses. C proteins of different genera share some mechanisms of action, but some C proteins have unique characteristics, probably reflecting viral adaptations to specific hosts or tissues.

### 10.1. Interferon Induction

While the IFN induction pathway (Figure 4, center) is a major target of paramyxoviral V proteins [24,128], C proteins also target it. SeV deficient for all four C proteins [SeV(4C−)] is a strong inducer of IFN expression ([129,130] due to its inability to prevent dsRNA generation [112]. On the other hand, the ectopic expression of SeV and BPIV3 C proteins inhibits phosphorylation and the dimerization of IFN regulatory factor 3 (IRF3) by TANK-binding kinase 1 (TBK-1) induced by foreign stimuli, such as Newcastle disease virus infection, poly(I:C) treatment, 5′-triphosphate containing RNA or infection with SeV DI particles [130,131,132]. This indicates that the respirovirus C protein may directly interfere with the signal transduction cascade leading to type-I IFN production, but the direct interaction partner has not been identified.

The C proteins of the morbilliviruses MeV, RPV and PPRV also interfere with IFN induction [73,133,134]. However, the phosphorylation, dimerization and nuclear translocation of IRF3 are not affected in cells expressing the MeV or RPV C proteins. On the other hand, the nuclear localization of MeV C is essential for the efficient inhibition of IFNβ induction, suggesting the inhibition of processes involved in *IFNB* gene transcription occurring in the nucleus [73]. Notably, MeV vaccine strain C proteins, which have a mutation in the NLS and, therefore, low nuclear accumulation, have a reduced ability to inhibit *IFNB* transcription [73]. A recent proteomic analysis of MeV C protein interaction partners revealed a weak interaction with the p65 subunit of the nuclear factor kappa B (NF-κB) transcription factor [135], which, in addition to IRF3, is part of the *IFNB* enhanceosome [136]. While the MeV growth was similar in parental and in p65 knockout cells, growth of the MeV-C^KO^ mutant was significantly enhanced, suggesting that the blocking of p65 by MeV C enhances viral replication [135]. Finally, a recent study suggested that the PPRV C protein can block *IFNB* promoter activation when a constitutively active form of RIG-I, or its downstream adapter mitochondrial antiviral signaling (MAVS), is expressed but not when downstream signaling components are overexpressed [137]. This suggests that the PPRV C protein, in contrast to the MeV and RPV C proteins, may target a step in the beginning of the signal transduction cascade.

### 10.2. Interferon Signaling

Upon the binding of IFNα or IFNβ to the type-I IFN receptor complex (IFNAR1/2), a signaling cascade involving receptor-associated tyrosine janus kinases JAK1, JAK2 and TYK2 leads to phosphorylation of the transcription factors STAT1 and STAT2 (Figure 4, IFN signaling). These then associate with IRF9 to form the IFN-stimulated gene factor 3 (ISGF3) complex, which drives the expression of several hundred IFN-stimulated genes (ISGs) under control of the IFN-stimulated response element (*ISRE*). These genes encode proteins with direct antiviral properties, as well as regulators (activators and suppressors) of the IFN response signaling pathways [138].

Like many other viruses, paramyxoviruses target the IFN-signaling cascade and thereby prevent ISG expression in infected cells. Many paramyxoviruses have evolved more than one mechanism that inhibit IFN signaling. Most importantly, V proteins of paramyxoviruses, and, to some extent P proteins, block IFN signaling by targeting STAT1, STAT2, and the janus kinases [25,26,27,28,128]. V proteins bind and block STAT1 via different mechanisms. While some V proteins, such as those of rubulaviruses, target STAT1 for proteasomal degradation [25,26,27,28], others, like morbillivirus V proteins, block its nuclear translocation [86,139]. Morbillivirus V proteins, in addition, bind to STAT2, and their zinc-binding carboxy-terminal domain (V_CT_) mediates this interaction [140]. In addition, SeV C proteins suppress IFN signaling, indicating how crucial efficient blocking of the IFN response is for this virus [141].

The paramyxovirus C proteins also contribute to silencing the IFN response. SeV C protein binds STAT1 [142,143], preventing both phosphorylation and dephosphorylation of its residue Y701 that is critical for activation [144,145]. Interestingly, different SeV C proteins act through two different mechanisms. Whereas the long proteins C’ and C induce STAT1 degradation [143,146,147], the shorter Y1 and Y2 proteins efficiently block STAT1 phosphorylation without inducing degradation [66,146,147,148].

The co-crystal structure of parts of the SeV C protein with STAT1 (PDB 3WWT; Figure 3A) [59] allows to infer the mechanism underlying this inhibition. The C protein carboxy-terminal globular domain binds to the amino terminal STAT1 domain (ND) via charged and hydrophilic interactions along nearly parallel alpha-helices. Unphosphorylated STAT1 forms homodimers or heterodimers via ND interactions, and this dimerization and a conformational change between the subunits lead to the exposure of the Y701 phosphorylation site to the IFNAR–JAK complex [149]. The crystal structure suggests that SeV C locks STAT1 homodimers and STAT1-STAT2 heterodimers in a conformation unfavorable for phosphorylation [59,150].

While this mechanism depends on the interaction of SeV C proteins with STAT1, additional mechanisms independent of this interaction contribute to the blocking of IFN signaling [151]. By binding to the cytoplasmic tail of IFNAR2, the SeV C protein blocks activation of the janus kinases JAK1 and TYK2 [152]. Notably, this additional mechanism to prevent STAT phosphorylation is conserved across respirovirus C proteins [152,153] and may require the amino terminus [154], while STAT1 binding is specific for the SeV C protein and involves the carboxy-terminus [59].

*Morbillivirus* C proteins, as exemplified by MeV and RPV, also interfere with IFN signaling by blocking STAT phosphorylation [155,156]. However, a direct comparison of the antagonistic activities of P, V and C proteins suggests that V is the major morbillivirus antagonist of IFN signaling [25,26,27,28]. In contrast, the activity of the C protein is much lower and may vary between different virus strains [155,156,157]. It is also unknown whether morbillivirus C proteins target IFNAR or JAKs similar to respirovirus C proteins to prevent STAT phosphorylation. Direct interactions of morbillivirus C proteins with either STAT1 or STAT2 have not been detected [156,157].

### 10.3. Inflammation

Inflammatory responses are critical for viral clearance but can also lead to severe pathology in the form of a cytokine storm or cytokine release syndrome. These side effects are reported for numerous respiratory pathogens, such as highly pathogenic influenza A viruses and SARS-CoV-2, as well as pathogens causing hemorrhagic fevers, such as the Ebola virus [158,159,160]. They are mediated by the excessive production and release of chemokines; IFNs and proinflammatory cytokines such as interleukin 6 (IL-6), IL-1β and IL-18. The transcriptional upregulation of IL-1β and IL-18 during RNA virus infections occurs after viral RNA sensing by RIG-I and MDA-5; however, these cytokines are expressed as inactive pro-forms, which require further activation (Figure 4). This activation is mediated by inflammasomes and inflammasome-associated caspases.

For the recognition of RNA viruses, NLR family pyrin domain containing 3 (NLRP3) is the main inflammasome component [161]. The NLRP3 inflammasome can be activated by viral PAMPs but also by other stimuli (or danger-associated molecular patterns), including uric acid crystals and reactive oxygen species [162,163]. Moreover, nitric oxide (NO), which is generated upon viral infection by inducible nitric oxide synthase (iNOS), is an important regulator of the NLRP3 inflammasome [164]. The expression of iNOS is stimulated by various transcription factors, including NF-κB and IFN-γ-activated STAT1 homodimers, in response to viral infections [165,166].

The NiV C protein suppresses cytokine and chemokine expression in infected endothelial cells [91,167]. Infection with C protein-deficient NiV (NiVΔC) led to increased chemokine and cytokine expression, including IL-1β, and, consequently, reduced pathogenesis compared to wild-type NiV infection in a hamster model [91]. The transcriptional upregulation of IL-1β by NiVΔC may be a direct consequence of increased RIG-I/MDA-5 activation by this virus. However, evidence for the inflammasome-mediated activation of IL-1β is missing. Notably, NiV is a hemorrhagic fever virus not associated with a cytokine storm [168].

The SeV C protein affects NO generation by limiting the dsRNA levels in infected cells, which otherwise would trigger an IFN response and lead to the upregulation of iNOS [169,170]. This may, in turn, affect inflammasome activation and the activation of proinflammatory cytokines. A more direct effect on the NLRP3 inflammasome has been reported for the SeV V protein: V directly interacts with NLRP3 and inhibits its assembly into a functional inflammasome [171]. A similar function was reported for the HPIV3 C protein [172]. The HPIV3 C protein binds to NLRP3 and directs it towards proteasomal degradation, thereby disabling inflammasome assembly. Intriguingly, blocking the NLRP3 inflammasome seems so important for respiroviruses that the C protein has taken over this function from the V protein in V-deficient viruses. In summary, paramyxoviral C proteins can directly bind to, and interfere with, the function of several cellular proteins required for IFN induction, IFN signaling and inflammation. These C protein tasks are carried out in addition to those supporting efficient and accurate viral replication.

## 11. C Proteins Support Viral Particle Assembly and Budding

Another task performed by C proteins during the paramyxovirus replication cycle is the support of particle assembly and budding. It was recognized early that the matrix (M) protein is essential to form and release new functional virus particles [173,174,175,176]. On the other hand, the M proteins of viruses from different subfamilies have distinct functional characteristics. Those of *Orthoparamyxovirinae* and *Avulavirinae* support the budding of virus-like particles (VLPs) that are generated in the absence of any other viral protein or a viral infection, proving that the M protein drives viral budding [177,178,179,180].

In the subfamily *Rubulavirinae*, however, M protein expression alone does not support the efficient budding of VLPs [181,182]. For this subfamily, the co-expression of other structural proteins (H, F and N) is required to achieve particle formation efficiencies comparable with a viral infection. The fact that co-expression of the glycoproteins H and F, as well as proteins of the RNP, modulate the budding of viruses of different *Paramyxoviridae* genera indicates that the M protein is not solely responsible for the budding process [178,181,182,183,184,185,186].

Notably, for viruses of the genera *Respirovirus* and *Henipavirus*, C proteins are involved in viral budding (Figure 4) [183,187]. The co-expression of the SeV C protein with other structural proteins enhances the release of particles in a VLP system [183]. Closer examination of the involvement of SeV C in budding identified the host protein ALIX as an interaction partner of the SeV C protein [188]. ALIX connects the ESCRT-1 protein complex with the ESCRT-3 complex [189,190,191]. These complexes facilitate protein transport through the multivesicular body and endosomal sorting [192,193].

Recently, the crystal structure of the carboxy-terminal domain of the SeV C protein (Y3) in a complex with the BRO1 domain of ALIX (PDB 6KP3) was resolved at 2.2 Å [61]. Within this structure, a LXXW motif in the SeV C protein interacts with ALIX (Figure 3B), and tryptophan is essential for this interaction [61]. However, for efficient C protein-induced budding, an amino-terminal peptide of 23 residues is also required for the membrane anchoring of C [188,194,195]. Therefore, only the C’ and C proteins are efficient inducers of SeV budding, whereas the Y1 and Y2 proteins are not [194]. Notably, the LXXW motif is conserved among respiroviruses (Figure 3C), and the HPIV1 C protein interacts with ALIX as well [196].

These data suggest that respiroviruses utilize the ESCRT pathway for budding. Indeed, interference with this pathway results in a decreased release of Sendai-VLPs [188,197] and reduced HPIV1 titers [196]. On the other hand, redundancies in the functions of viral M and C proteins, as well as the different ESCRT systems, suggest that respiroviruses rely on multiple parallel mechanisms for efficient particle release [197,198]. Interestingly, C proteins of some morbilliviruses, namely MeV, CDV and PMV, possess a LXXW motif (Figure 3C). However, MeV C protein does not bind ALIX, and the virus particle release is inefficient and seems independent of the ESCRT pathway [188,199], suggesting that the release of morbillivirus particles may follow a different mechanism.

The NiV C protein interacts with ESCRT factor Tsg101 [187]. This interaction depends on two residues: W103 and L104, which are conserved among henipaviruses (Figure 3D). This WL dipeptide is also conserved in some respirovirus C proteins (Figure 3D), but the evidence for an interaction of respirovirus C proteins with Tsg101 is missing.

In conclusion, the budding mechanisms of paramyxoviruses differ between genera. Respiroviruses and henipaviruses use the ESCRT system for efficient particle release, but the corresponding C proteins have different supporting roles. In contrast, particle release from morbillivirus-infected cells is inefficient and independent of the ESCRT system [199]. These viruses rely on the formation of intercellular fusion pores for cell-to-cell spreading [200,201,202], and it is unclear whether C proteins participate in these processes.

## 12. Concluding Remarks

The small C proteins expressed by viruses of three genera of the *Orthoparamyxovirinae* subfamily control a wide range of viral and cellular processes. All of them modulate the viral polymerase activity, thereby assuring proper RNA synthesis and avoiding the generation of aberrant replication products, which would trigger innate immune responses. In addition, some C proteins directly interfere with the function of specific innate immunity proteins. Finally, some C proteins support efficient viral budding.

While C proteins were initially categorized as nonstructural or accessory, we now understand better why they are so important for viral pathogenesis. Since C protein-deficient viruses are highly attenuated, they could serve as novel live-attenuated vaccines. In addition, novel antiviral therapeutics may target some C protein functions, to be used in combination with existing fusion and polymerase inhibitors [203,204,205]. In consideration of the multiple functions of paramyxovirus C proteins in the control of virus replication and of the host innate immune response, we propose that C should stand for “control”.

## Figures and Tables

**Table 1:** Overview of C protein-expressing paramyxoviruses.

Genus	Species	Abbreviation	Genome Sequence	C Protein Sequence		Reference
			(GenBank)	(GenBank)	(UniProt/UniParc)	
Aquapara-myxovirus	Oncorhynchus aquaparamyxovirus	PSPV	MH900516.1	AYN62575.1	I1TLL1	[29]
	Salmo aquaparamyxovirus	ASPV	NC_025360.1	YP_009094145.1	B2BX73	[30]
Henipavirus	Cedar henipavirus	CeV	NC_025351.1	YP_009094083.1	J7H4I1	[31]
	Hendra henipavirus	HeV	NC_001906.3	NP_047109.1	O55779	[32]
	Nipah henipavirus	NiV	NC_002728.1	NP_112024.1	Q997F1	[33]
Jeilongvirus	Beilong jeilongvirus	BeV	NC_007803.1	YP_512248.1	Q287X7	[34]
	Jun jeilongvirus	J-V	NC_007454.1	YP_338079.1	Q49HN8	[35]
Morbillivirus	Canine morbillivirus	CDV	NC_001921.1	NP_047203.1	P06941	[36]
	Cetacean morbillivirus	CeMV	NC_005283.1	NP_945026.1	Q709E7	[37]
	Feline morbillivirus	FeMV	NC_039196.1	YP_009512960.1	UPI000259F006	[38]
	Measles morbillivirus	MeV	NC_001498.1	NP_056920.1	Q9YZN9	[39]
	Phocine morbillivirus	PMV	NC_028249.1	YP_009177600.1	P35940	[40]
	Rinderpest morbillivirus	RPV	NC_006296.2	YP_087122.1	P35948	[41]
	Small ruminant morbillivirus	PPRV	NC_006383.2	YP_133824.1	Q5ZER5	[42]
Narmovirus	Mossman narmovirus	MoV	NC_005339.1	NP_958051.1	Q6WGM3	[43]
	Myodes narmovirus	BaVV	NC_055167.1	YP_010085011.1	N/A	[44]
	Nariva narmovirus	NarV	NC_017937.1	YP_006347585.1	B8XH61	[45]
	Tupaia narmovirus	TPMV	NC_002199.1	NP_054693.1	Q9WS38	[46]
Respirovirus	Bovine respirovirus 3	BPIV3	NC_002161.1	N/A	N/A	[47]
	Caprine respirovirus 3	CPIV3	NC_028362.1	N/A	N/A	[48]
	Human respirovirus 1	HPIV1	NC_003461.1	NP_604436.1	Q8QT30	[49]
	Human respirovirus 3	HPIV3	NC_001796.2	NP_599251.1	UPI0000161E9C	[50]
	Murine respirovirus	SeV	NC_001552.1	NP_056872.1	O55527	[51]
	Porcine respirovirus 1	PPIV1	NC_025402.1	YP_009094446.1	S5LSI4	[52]
	Squirrel respirovirus	GSqRV	LS992584.1	SYZ47172.1	A0A383S9W5	[53]
Salemvirus	Salem salemvirus	SalV	NC_025386.1	YP_009094334.1	Q9IZB9	[54]

N/A: no sequence accession number available in GenBank or UniProt; C protein ORFs of these viruses were identified in the genome sequence and translated. Prototype species NiV, MeV and SeV in bold face.

## Data Availability

Not applicable.
